# The Treatment of Heterotopic Ossification With a Dual Mobility Total Hip Replacement System: A Case Report

**DOI:** 10.7759/cureus.23977

**Published:** 2022-04-09

**Authors:** Sarthak Parikh, Collin Tacy, Osmanny Gomez, Arturo Corces

**Affiliations:** 1 Department of Orthopedic Surgery, Larkin Community Hospital, Miami, USA; 2 Department of Osteopathic Medicine, Nova Southeastern University Dr. Kiran C. Patel College of Osteopathic Medicine, Fort Lauderdale, USA

**Keywords:** trauma, orthopedic spine surgery, dual radius system, total hip replacement, heterotopic ossification

## Abstract

Heterotopic ossification (HO) is the formation of bone within extraskeletal soft tissue. The development of mature lamellar bone within soft tissues can be acquired in cases like trauma. Clinical manifestations of HO primarily include pain at the site of the extraskeletal ossification and limited range of motion or function when it involves a joint. This case report presents a 56-year-old man with severe HO. His past medical history included a traumatic hip dislocation in 1996. He denied any other past medical, family, or surgical history. This patient had severely limited range of motion and difficulty performing activities of daily living like going up and down the stairs and getting up from a seated position. After failing conservative therapy with non-steroidal anti-inflammatory drugs (NSAIDs) and physical therapy, a non-cemented dual mobility hip replacement system was used to treat this patient. A non-cemented dual mobility hip replacement system was chosen because the patient had significant bone loss and was relatively young. The dual mobility system significantly reduces the risk of dislocation and is a good option for younger patients who require more stability in their hips. The patient progressed well with a full range of motion and no pain. There was no evidence of HO recurrence. Treatment of HO with a total hip replacement, let alone a dual mobility system, is not prevalent throughout the literature. Furthermore, cemented total hip arthroplasty has been associated with increased recurrence of HO, which is why we elected to use a non-cemented technique. Osteoplasty is typically the mainstay of treatment for HO. The purpose of this case report is to introduce an incident of HO treated with a non-cemented dual mobility system and emphasize its use in young, middle-aged, or active patients who have bone loss and require increased stability.

## Introduction

Heterotopic ossification (HO) is the formation of bone within extraskeletal soft tissue. The development of mature lamellar bone within soft tissues can be acquired or arise in congenital conditions. The two congenital conditions that are associated with HO are fibrodysplasia ossificans progressiva and progressive osseous heteroplasia, both being rare conditions that indicate at least a partial genetic association for the development of bone within soft tissue [1]. The most common etiology of HO is acquired and is secondary to musculoskeletal trauma, spinal cord injury (SCI), or traumatic brain injury (TBI) [2]. HO commonly arises following musculoskeletal trauma in the form of joint arthroplasty or traumatic fractures. The most common fracture that leads to the development of HO is an acetabular fracture [3]. Despite these etiologies, the exact pathophysiology of HO is unknown.

The incidence of HO varies depending on the mechanism of injury but generally ranges from 10% to 53% following SCI or TBI, with 40% following joint arthroplasty (specifically total hip arthroplasty (THA)) [2]. Some evidence suggests that local and systemic inflammatory cytokines may be implicated in the development of HO, especially following traumatic injury [1]. Two factors that may stimulate the cascade of ectopic bone formation following trauma include local tissue ischemia and activation of pluripotent stem cells [3]. Local inflammatory mediators, specifically bone morphogenetic protein (BMP), may also have a role in the conversion of pluripotent stem cells to osteoblasts within soft tissue [3]. Similarly, inflammatory mediators (transforming growth factor-beta, insulin-like growth factor II, platelet-derived growth factor, interleukin-1, and interleukin-6) are upregulated following central nervous system injury in animals and lead to increased conversion of pluripotent stem cells to osteoblasts [4].

Clinical manifestations of HO primarily include pain at the site of the extraskeletal ossification and limited range of motion or function when it involves a joint. Other symptoms, such as numbness and weakness, may be present when the HO causes compression of surrounding neurovascular structures [3]. The severity of HO is based on its clinical presentation and the Brooker classification system, which separates HO into four categories (I, II, III, and IV) based on its radiographic findings after total knee arthroplasty (TKA) [5]. A progression in the scale is determined by measuring the distance of extra-axial bone growth between the pelvis and the greater trochanter of the femur.

Treatment of HO largely involves surgical intervention, as conservative measures with physical therapy and pharmaceutical intervention have very limited efficacy in improving patient symptoms and function [2]. Surgical intervention typically involves an osteoplasty and excision of heterotopic bone after the heterotopic bone has matured. Excision of heterotopic bone prior to maturation can result in an increased incidence of recurrence [6]. The degree of functional limitation should serve as a guide to management strategies, as the most common long-term sequela of HO involves limitations in mobility and joint function. Prophylactic measures with non-steroidal anti-inflammatory drugs (NSAIDs) may be helpful in preventing the formation of HO by inhibiting osteogenic differentiation. Bisphosphonates can also be used to induce osteoclast apoptosis and prevent calcification. Low-dose radiation has also been shown to reduce HO formation [7]. However, there is varying evidence to support many of these prophylactic regimens and each one must be carefully considered against possible patient risk factors.

According to the literature, surgical excision is the main operative technique for the treatment of HO. Furthermore, many instances of HO occur after THA [1,6,8-11]. The purpose of this case report is to describe an incident of HO as a result of previous traumatic hip dislocation that was treated with THA, specifically with a dual mobility cup. Although THA as a treatment for HO is not prevalent throughout the literature, it may be used as a treatment modality in conjunction with osteoplasty.

## Case presentation

**Figure 1:**
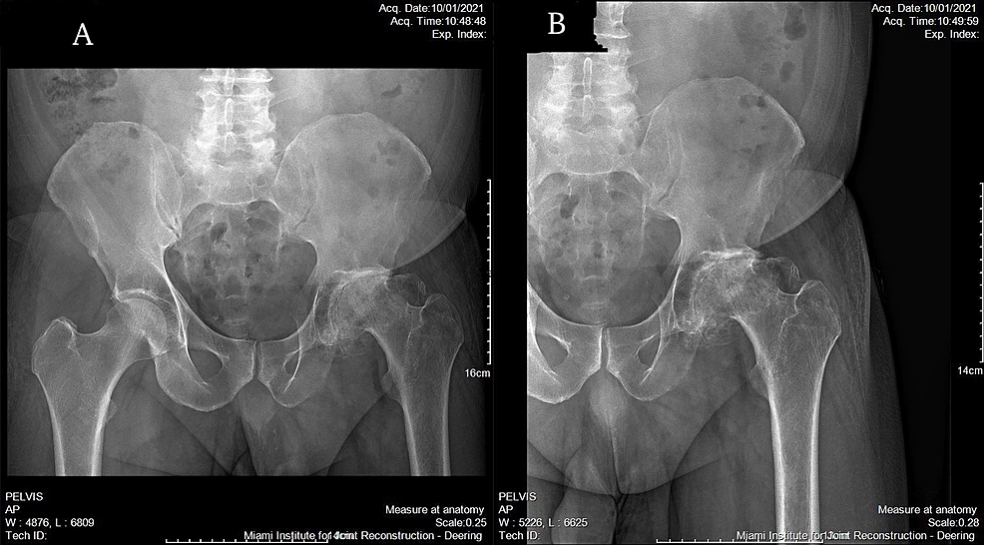
Preoperative anteroposterior radiographs of the (A) pelvis and (B) left hip.

**Figure 2:**
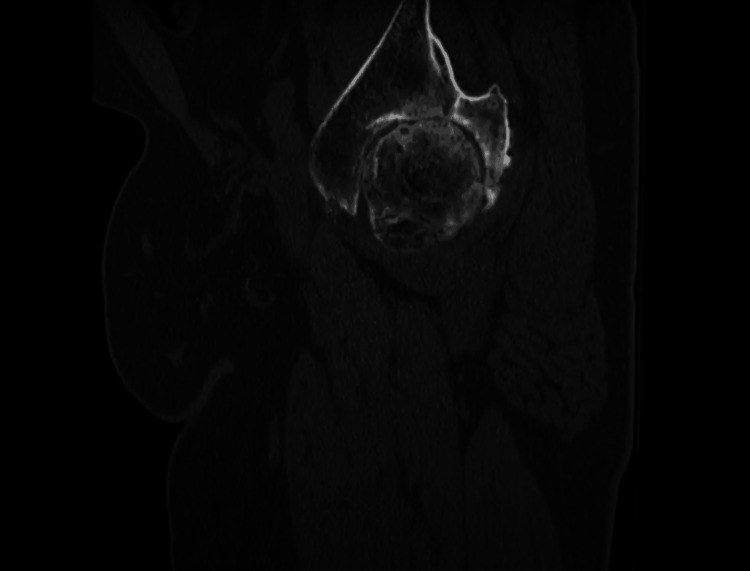
Sagittal CT scan of the left femoral acetabular joint.

**Figure 3:**
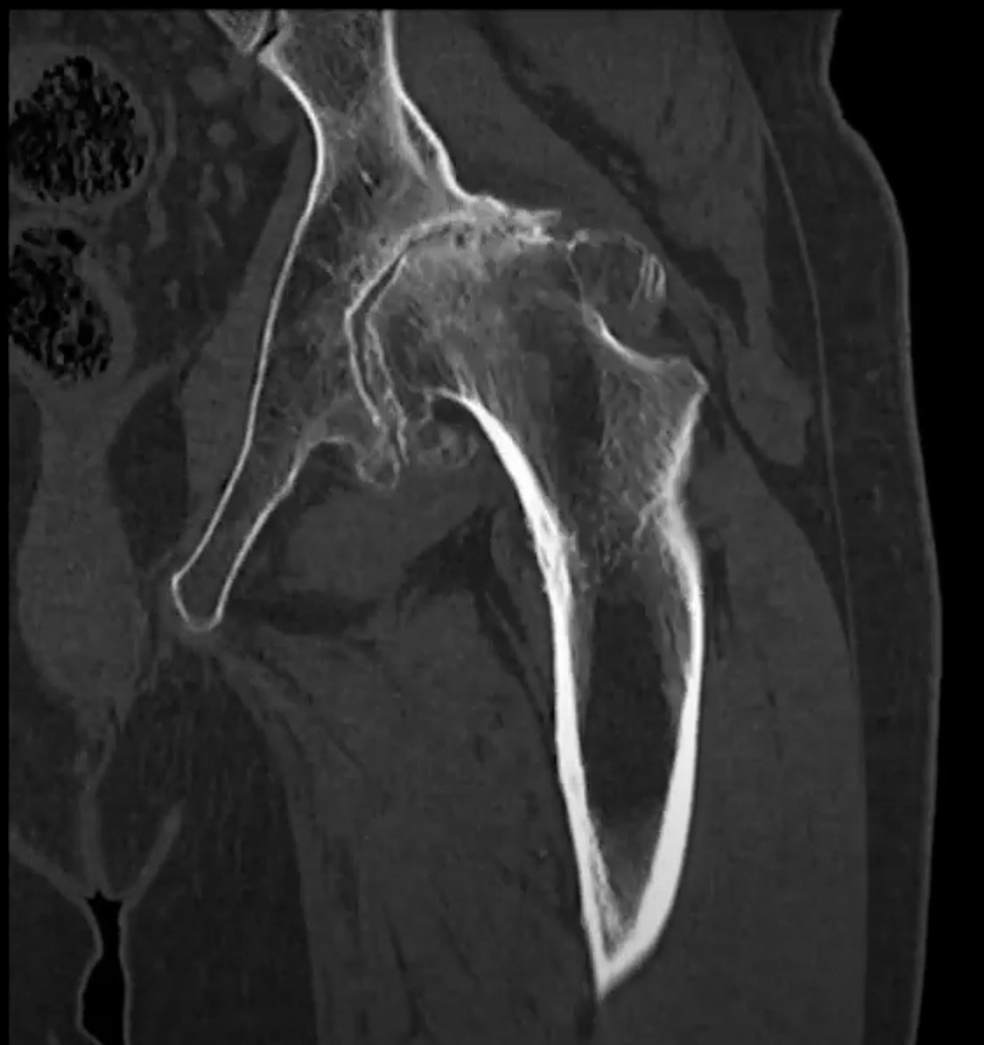
Coronal CT of the left hip and femoral acetabular joint.

**Figure 4:**
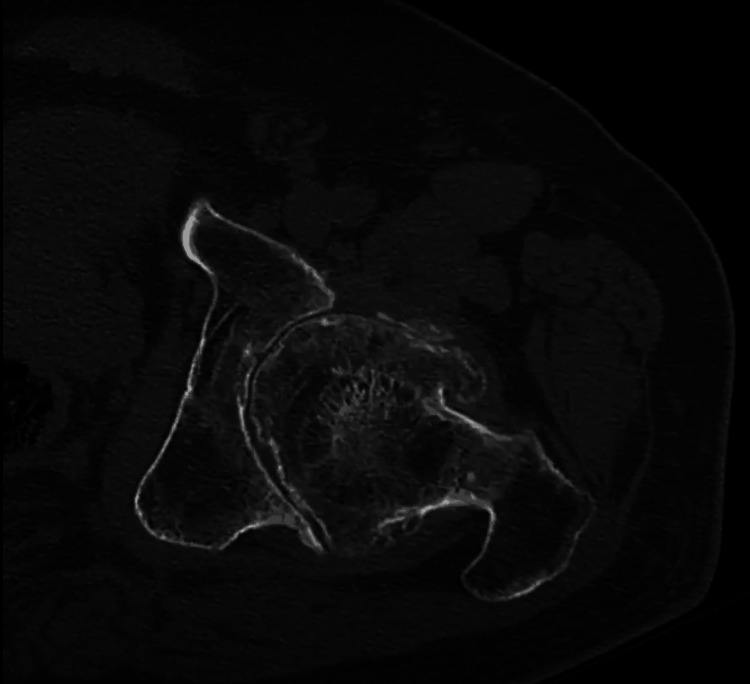
Axial CT scan of the left hip and femoral acetabular joint.

We present a case of a 56-year-old male who presented to an outpatient clinic with persistent left hip and groin pain localized to the left groin and greater trochanter. He was unable to ambulate more than two blocks without an assistance device. The patient had difficulty walking and performing normal activities of daily living. His movement was significantly limited by pain. He had no past medical or surgical history. His family history was unknown. The patient complained that he had difficulty performing activities like going up and down the stairs or getting up from a seated position. Inspection of the area showed no deformities or skin changes. Active and passive range of motion was severely limited in all planes. The exact ranges were not recorded. There was pain on flexion and internal rotation. The straight leg test was negative and there was no clinical or physical indication of pain originating from his back. The patient is an immigrant from Cuba who suffered a hip dislocation in 1996 because of a motor vehicle accident. The radiographic examination consisted of an anteroposterior pelvis radiograph and sagittal, coronal, and axial CT scans (Figures 1-4). These images demonstrated a severely arthritic left hip with osteophyte formation and extra-axial bone growth surrounding the femoral head and acetabulum. The left acetabular socket was deep into the ilioischial line, indicating coxa profunda.

**Figure 5:**
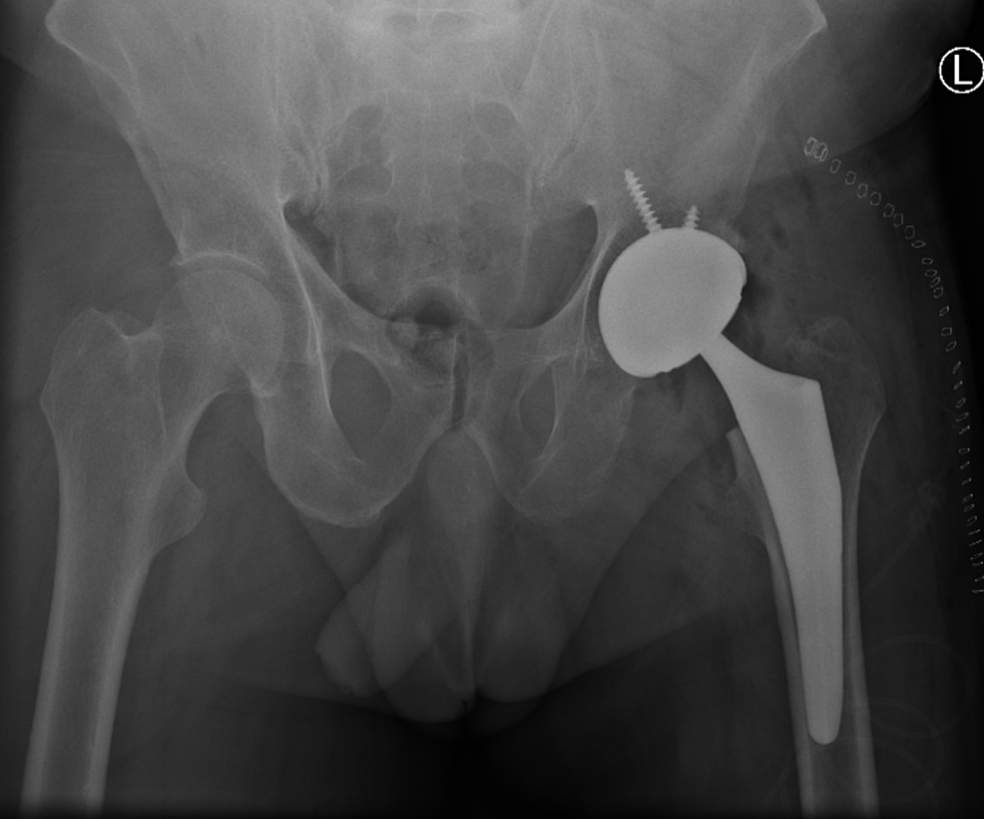
Postoperative anteroposterior radiograph of the pelvis after total hip replacement with a dual mobility system.

The patient’s hip has failed conservative treatment with anti-inflammatory medication and physical therapy. Therefore, the best treatment modality for this patient was a total hip replacement. Because significant bone was resected and the patient was at a higher risk for dislocation, a dual radius system was used. Because this patient was relatively young and significant bone was lost, a non-cemented dual radius system was used. Post-surgical treatment with anti-inflammatory medication and physical therapy improved pain and range of motion. No recurrence of HO or repeat dislocation has occurred. A postoperative radiograph was done (Figure 5).

## Discussion

The purpose of this case report is to emphasize the use of a non-cemented dual mobility THA system in patients with HO from traumatic dislocation, specifically in patients who have bone loss. THA may be used as a treatment for HO, but its use for HO is not prevalent throughout the literature. Instead, the literature discusses how THA is a risk factor for HO and how HO should be managed after THA [7,8,10-12]. Patients with HO resulting from traumatic dislocation are prone to osteoarthritis and severe bone loss. Therefore, these patients may benefit from a dual mobility THA instead of just osteoplasty. A dual mobility cup allows for enhanced stability due to its dual articulation capability [1,3]. By incorporating a mobile polyethylene layer between the prosthetic head and the acetabular shell, the dual mobility cup combines Charnley’s low-friction principle with the McKee-Farrar concept of an increased femoral head-to-neck ratio. This maximizes stability and results in a greater range of motion before impingement or dislocation [13-15]. A study by Hernigou et al. compares rates of dislocation in high-risk obese patients undergoing THA with either a dual mobility system or a standard cup. Their results showed a statistically significant reduction in dislocation in those who had a dual mobility or constrained (2%) liner rather than a standard cup (9%) at seven years of follow-up [16].

This patient had severe hip arthritis secondary to traumatic HO with no history of treatment. After failing conservative treatment, surgical intervention with a dual radius total hip replacement was best. This was implemented because of the patient’s severe bone loss and need for increased stability. A dual mobility hip system may be a better treatment modality than a traditional hip replacement for those requiring more stability and greater range of motion before dislocation. Furthermore, HO is more common in bilateral and cemented THA [17]. In our case, we elected to use a non-cemented technique on a unilateral hip to address possible risk factors [13]. HO is commonly treated with osteoplasty, but because this patient had severe osteoarthritis, a THA was the best treatment option. Recurrence of HO is not uncommon, specifically, in those with the removal of immature bony fragments [12]. Our patient, however, has continued to have normal follow-up and serial radiographs, which show no extraskeletal bone lesions. Nevertheless, some common concerns regarding dual mobility systems include fretting, corrosion, and long-term survivorship [13]. High-quality, prospective, comparative studies are needed to evaluate the longevity of these devices.

## Conclusions

The purpose of this case report is to illustrate a severe form of HO associated with dislocation and treated with a non-cemented dual radius hip replacement system to prevent further dislocation and injury. This patient had severe hip arthritis secondary to HO with no history of treatment. After failing conservative treatment, surgical intervention with a dual radius total hip replacement was best. This was implemented because of the patient’s severe bone loss and history of dislocation. A dual radius total hip replacement allows for enhanced stability as a result of its dual articulation capability. This results in a greater range of motion before impingement or dislocation. We elected to use a non-cemented technique on a unilateral hip to address possible risk factors for further recurrence. Our patient, however, has continued to have normal follow-up and serial radiographs, which show no extraskeletal bone lesions. Dual mobility hip replacements should be considered in those with a history of traumatic dislocations and concomitant HO.
